# Bloody Tumor of the Vagina Occurring during Labor

**Published:** 1856-03

**Authors:** J. K. Mason


### *Bloody Tumor of the Vagina occurring during labor.*

By J. K. Mason, M. D.

I have been induced to publish the following case, under the conviction that it is of very rare occurrence; not one of my professional brethren with whom I have conversed on the subject have ever seen one precisely similar, and in looking over various standard works on the subject of midwifery, I do not find any allusion to such an accident, nor in searching through the various journals to which I have access, have I seen such a case reported.

I report the case as it occurred, with its management, in the hope that it may prove practically useful to some who may hereafter be compelled to act under similar circumstances.

Was called to attend Mrs. W., æt. 34, in her first accouchement, a spare woman of a leucophlegmatic temperament and great laxity of muscular fibre. On examination I found the os uteri open to the extent of half a dollar, the bag of water presenting well, the child's head above the superior strait; as the pains were but feeble, the patient chilly, and the pulse perfectly unaffected, I felt certain that actual labor would not commence for some time, and took my leave, in order to attend to other duties, promising to return as soon as possible. In about two hours I again saw her; on examining, found the os uteri well opened, the waters evacuated, and the child's head nearly through the superior strait, presenting in the fourth position of Baudelocque, that is, with the occiput looking towards the right sacro-iliac synchondrosis, and the anterior fontanelle to the left acetabulum; the pains were not very active, but still the woman made loud complaints, and from temperament I imagine, seemed to suffer more than such feeble uterine contractions warranted.

She was ordered warm drinks and a stimulating injection, which opened her bowels; after this the labor became more active, and I began to hope that the affair would soon terminate without trouble, for the perineum and vaginal walls were more than commonly relaxed, too much so indeed, and therefore, though an occipito-posterior position, I assured myself that the occiput, the head being well flexed upon the breast, would clear the fourchette without much difficulty.

After the delay of an hour, during which time the uterine contractions were sufficiently active, I again touched, but was disappointed to find that the labor had not in the slightest degree advanced; the head was in the same position it had occupied two hours before, and keeping my hand in contact with it during a pain, I found that the uterine contractions produced no effect upon it. This state of things having continued for some time, I determined to apply the forceps and deliver without delay; this was done with great care and gentleness, but notwithstanding every precaution, the perineum was ruptured, giving way like a piece of wet paper. The child was alive and unhurt, but on making an after examinaticn I found another child's head presenting in the first position at the superior strait. With so favorable a position, and the external organs so thoroughly dilated, I anticipated no further trouble, and made up my mind to wait. After some time, the pains being active, I passed my hand gently into the vagina and discovered that the head was in the superior strait, but could not descend in consequence of a large tumor, which extended from the left external labium up to the presenting part, and apparently filled the whole vagina. It was evident that some vessel deeply seated, perhaps a varicose vein, (though none were apparent externally,) had given way, and was pouring out its contents between the tissues of the part.

The question for consideration now was, whether to open the tumor or to apply the forceps and attempt delivery, but I felt that previous to any further operation, it would be just to my patient to give her the benefit of a consultation. Dr. John Neill, with great promptitude and kindness, gave me his assistance, and after mature deliberation it was decided to have recourse to the forceps in preference to evacuating the tumor by means of the knife, for we considered if an incision were made before the head could descend by the natural efforts, a great deal of blood might be lost, whereas, by forced delivery, though the tumor would probably be ruptured, the head by its immediate pressure would arrest any further disposition to internal hemorrhage; the result I think incontestibly proves that the decision was a good one.

The patient being placed on her back, with the nates drawn to the edge of the bed, and well steadied by Dr. Neill, (who, as a precaution, kept his hand between the sphincter ani and the child's head,) I applied the forceps. Upon making firm traction the tumor burst, and its contents were spurted over us; after this the child was brought into the world with very trifling effort. Dr. Neill removed some clots from the cavity of the tumor, but all hemorrhage had entirely ceased. A compress was applied in the vagina, and after being placed carefully in bed, an opiate was administered, under the influence of which the patient slept comfortably.

The accident which most we feared in this case was sloughing of the walls of the vagina, but I am happy to say that no such consequence occurred. The lochiæ were profuse and of bad odor for some little time, but they soon became natural both in quantity and quality, and the wound in the perineum, though extending down to the sphincter ani, by no other treatment than perfect rest, with the limbs kept well together, and the application of poultices, healed perfectly; great tenderness of the parts was of course complained of for some time, but the patient made a perfect recovery, had abundance of milk, and both the children were healthy and vigorous.

I am perfectly aware that many of the profession, particularly those whose practical experience has not been extensive, will say: Doctor, how was it that this perineum was torn? surely there must have been some carelessness or mismanagement in this case. I can only say that it was not so, and can only account for the accident by the fact which I before stated, viz., that the tonicity of the muscular fibre was so poor that the parts gave way almost without an effort. In the same way I am inclined to account for the rupture of the coats of the internal vessel, which caused the bloody tumor, for the force applied was exceedingly trifling, and the vagina and external parts so perfectly relaxed that the perineum did not make the slightest resistance. Indeed I am disposed to think that such are the cases in which rupture of the perineum generally takes place, for I

have never seen the misfortune occur to strong muscular women of powerful fibre where the parts were greatly distended and the resistance appeared to be tremendous. Be it as it may, I have described the case as it occurred, and can only say to those who think that a perineum should never be torn, wait and see.

The celebrated Dr. Simpson, of Edinburgh, whose talents no man denies, and whose experience has been most enviably extensive, makes the following remarks on this very subject.

"These lesions are not, as has been alleged, necessarily the result of mismanagement; but they occur constantly in practice, despite every modification of management, and in cases also in which no kind of management has been adopted.

"The proper management and support of the perineum no doubt modifies and diminishes this form of perineal lesion, but it fails far more frequently than is generally believed in entirely preventing it."

Stitches in the torn perineum would in this case have been worse than useless, as they would only have inflicted unnecessary pain and given additional uneasiness, it being evident, I think, that the profuse discharges must have rendered union by the first intention impossible.

